# Marine Biotoxins in Crustaceans and Fish—A Review

**DOI:** 10.3390/toxins17120589

**Published:** 2025-12-09

**Authors:** Anna Madejska, Jacek Osek

**Affiliations:** Department of Microbiology of Food and Feed, National Veterinary Research Institute, Partyzantów 57, 24-100 Puławy, Poland; josek@piwet.pulawy.pl

**Keywords:** marine biotoxins, crustaceans, fish, monitoring, human health, detection methods

## Abstract

In recent years, there has been an increase in the consumption of seafood such as shellfish and crustaceans due to their pleasant taste and nutritional value. Fish are also a crucial part of a healthy, balanced diet. However, the consumption of these products may cause food poisoning through marine biotoxins. In recent years, several legal acts have been published by the European Commission to regulate toxin limits and describe their reference analysis methods. Commission Regulation (EC) No. 853/2004 established the maximum contents of marine biotoxins only in bivalve mollusks. Although other groups of marine organisms such as crustaceans (crabs, shrimps, and lobsters) and fish are not included in the EU rules for toxin monitoring, they may still be vectors of marine biotoxins for humans. Due to this, there is an urgent need for studies regarding the occurrence of marine biotoxins in non-bivalve seafood organisms and their potential influence on public health. In this review, the most important cases of accumulation of marine biotoxins in crustaceans and fish in recent years are described.

## 1. Introduction

**Figure 1:**
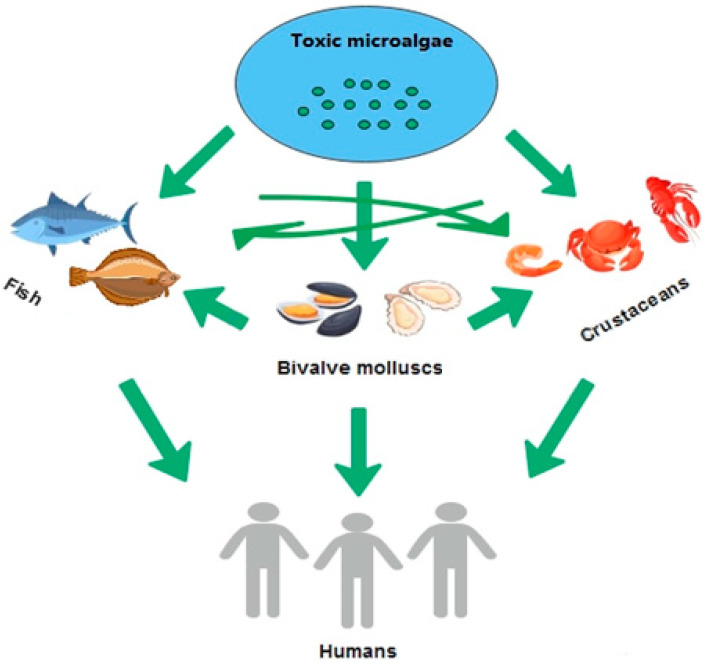
Exposure of humans to marine toxins through different vectors.

Marine biotoxins, also called phycotoxins, are a group of toxic compounds produced mainly by the massive growth of phytoplankton during harmful algal blooms (HABs) [1,2,3]. The occurrence of HABs is connected to the overgrowth of algae, which is often called ‘red tides’ due to change in color of the water surface. The formation of marine biotoxins is dependent on the presence of favorable conditions for their growth, i.e., the availability of nitrogen and phosphorus, appropriate water temperature, salinity, and CO_2_ concentration. Among the roughly 5000 existing species of marine algae, more than 100 may occur in very large amounts and produce toxins that are harmful for animals and humans [3]. Species of algae from the genera *Noctiluca scintillans* and *Skeletonema costatum* only change the color of water and cause the death of fish and other marine animals, but algae of *Alexandrium, Gymnodinium*, *Dinophysis*, and *Pseudo-nitzschia* are mainly responsible for poisoning humans. Aquatic organisms absorb marine biotoxins through food, and the biotoxins accumulate in different parts of the body, thus posing a risk for the consumers’ health [1,2]. Potential vectors of marine toxins are bivalve mollusks (e.g., blue mussels, Japanese carpet shell, Pacific oysters, and razor clams) [2,3], crustaceans (crabs, lobsters, shrimps) [1,4], and fish (e.g., northern anchovies, globefish, Pacific sardines, puffer fish) [1,3] (Figure 1). Mussels can filter 20 L of water per hour and harmful algal blooms may produce several million algal cells per liter of water [3].

The beneficial properties of marine biotoxins should also be emphasized. This diverse group of naturally occurring metabolites consists of very complex molecules that, despite their toxic properties, are also widely used in many fields such as pharmaceutical and biotechnology industries [5]. It is known that marine biotoxins possess anti-cancer, anti-inflammatory, anti-bacterial, and neurodegenerative properties and may be effective in the treatment of diabetes and hypertension.

The groups of marine organisms responsible for biotoxin poisoning were not expanded and remained the same since Regulation No. 853 regarding the limits for marine biotoxin in live bivalve mollusks established in 2004 [6]. The development of analytical methods has led to the detection of new toxin groups. A lot of them are called ‘emerging toxins’, which are involved with new occurrences that result from rising water temperatures and escalating coastal eutrophication. Furthermore, climate and hydrographic changes can also affect phytoplankton species distribution. Despite the implemented marine biotoxin monitoring programs, there is still a group of toxins without an established regulated limit [7]. Taking into account the symptoms of illness after the consumption of contaminated food, the most important biotoxins are classified as amnesic shellfish poisoning (ASP), azaspiracid poisoning (AZP), toxins causing paralytic shellfish poisoning (PSP), diarrheic shellfish poisoning (DSP), and yessotoxin (YTX), which are regulated by the European Union legislation, and emerging toxins that are not regulated such as neurotoxic shellfish poisoning (NSP), cyclic imines (CIs), ciguatera fish poisoning (CFP), and puffer fish poisoning caused by tetrodotoxin (TTX) [3,8] (Table 1).

Based on chemical properties, marine biotoxins can be classified as hydrophilic or lipophilic toxins [1,3,9]. In Europe, there are two separate groups of legislated hydrophilic toxins: saxitoxin (STX) and domoic acid (DA), along with their derivatives. The STX group is the most complex and comprises the following: the STX group (STX, decarbamoyl saxitoxin (dcSTX), the C group, N-sulfocarbamoyl-gonyautoxins 1–4, (C1, C2, C3, and C4)); GTX group, gonyautoxins 1–6 (GTX1, GTX2, GTX3, GTX4, B1 (GTX5), and B2 (GTX6)); decarbamoyl gonyautoxins 1–4 (dcGTX1, dcGTX2, dcGTX3, and dcGTX4); and neosaxitoxin (NEO, dcNEO). The main DA isomers comprise epi-domoic acid (epi-DA) and iso-domoic acids A, B, C, D, E, F, G, and H (iso-DA A-H) (1). Lipophilic marine toxins consist of four groups with different chemical structures: okadaic acid (OA), azaspiracids (AZAs), pectenotoxins (PTXs), and yessotoxins (YTXs), as well as their derivatives. Among them, a total of 13 analogs are registered in the European Union: OA and its derivatives, dinophysistoxins DTX-1, DTX-2, and DTX-3, AZA-1, AZA-2, AZA-3, PTX-1, PTX-2, YTX, 45-OH-YTX, homoYTX, and 45-homoYTX.

New emerging toxin groups often occur not only in bivalve mollusks but also in fish, e.g., puffer fish and Ciguatoxic fish [10,11,12]. Symptoms of poisoning mainly include acute gastrointestinal manifestations with diarrhea, nausea, vomiting, stomachache, and neurological disorders such as muscular paralysis, dizziness, memory loss, and respiratory difficulty [3]. Despite the fact that Regulation (EC) No. 2017/625 includes procedures for monitoring and sampling intended not only for live bivalve mollusks, but also marine gastropods, tunicates, and echinoderms, these organisms are frequently ignored in surveillance programs [7]. Similarly, crustaceans and fish may be vectors of marine biotoxins, but they have never been inspected for the content of these compounds [4,8].

There are a lot of scientific reports and documented studies including cases of detecting marine biotoxins in aquatic organisms other than shellfish [4]. Several human outbreaks of poisoning involving marine biotoxins in crustaceans have been reported [13,14,15]. However, there are no established regulations regarding sampling plans, regulatory limits, or reference methods of detection for organisms other than bivalve mollusks. For this reason, it is very important to collect data about biotoxins in crustaceans and fish.

The aim of this review is to present the most important information regarding the occurrence of marine biotoxins in non-bivalve organisms such as crustaceans (crabs, lobsters, shrimps) and fish and also to assess their risk to human health.

### Seafood Production and Global Market

Irrespective of the negative impact on human health, poisoning through marine biotoxins can cause serious economic consequences. The main sectors affected by harmful algal blooms are the fish and seafood trade, public health, tourism, and recreation. According to the data from a report by the Food and Agriculture Organization (FAO) of the United Nations, ‘The State of World Fisheries and Aquaculture 2022’, the global production of fish and seafood in 2022 was at a record high level, and this sector will play an increasingly important role in food supply and food safety worldwide [16]. Global seafood consumption (including fish) reached 20.2 kg per person in 2020, more than twice that in the 1960s. It is estimated that by 2030, consumption will increase to 21.4 kg per person. In 2020, the total amount of fish and seafood caught was at 90.3 million tons, and the total amount from direct seafood sales was USD 406 billion, while the production of crustaceans was at 11.2 million tons, worth USD 81.5 billion. Compared to the previous report, in 2018, the production of crustaceans was at 9.4 million tons for aquaculture worldwide, which corresponds to about USD 69.3 billion [17]. Among this group of organisms, the most commonly produced crustacean in the world is shrimps, followed by crabs and lobsters [18,19]. The increasing consumption of seafood dishes and common consumption of raw or lightly processed crustaceans can cause the transmission of harmful algal toxins, resulting in food poisoning of various severity, ranging from mild to even causing death [20]. Fish, desirable sources of valuable nutrients such as protein, omega 3-fatty acids, and vitamins, are also exposed to harmful algal blooms.

## 2. Challenges in the Detection of Marine Biotoxins

The search for new methods to determine marine biotoxins and improve the current ones is extremely important, not only to ensure consumers’ safety but also due to their pharmacological properties and clinical application in the form of new drugs and therapeutic paths [21]. The growing number and types of biotoxins and food matrices indicate the need to develop new procedures in the monitoring of production areas of seafood and fish. At present, the methods used to determine marine biotoxins can be divided into biological, functional, and chemical assays. Also, numerous processes of extraction and detection of marine biotoxins are crucial for determining the quality and safety of seafood. Advanced chemical methods, such as high-performance liquid chromatography (HPLC) with UV or FLD detection and liquid chromatography–mass spectrometry (LC-MS), as well as novel techniques like nanomaterials and biosensors, are used for the effective detection and quantification of biotoxins in seafood [3,9,22]. Due to the specific identification procedures for each marine biotoxin, the obtained results correspond to specific concentrations of the determined compounds and can be calculated into toxic equivalents by using appropriate conversion factors. The numerous studies conducted in this field are essential for solving the emerging problems related to the detection of these heterogeneous compounds in different food matrices.

The first official method for the analysis of the paralytic shellfish poisoning (PSP) toxin was a mouse bioassay (MBA) [3]. MBA is a biological method based on the intraperitoneal injection of a seafood extract into the mouse, and then monitoring the survival or the time it takes the animal to die to determine the presence and concentration of the toxin [9]. The biotoxin level is presented in Mouse Units (MU) multiplied by the dilution factor and, if necessary, by a weight correcting factor, giving finally the concentration of PSP in MU/100 g. To convert MU into μg of STX equivalent, the result is multiplied by a conversion factor, which is regularly controlled and calculated by each laboratory. In addition to the ethical concerns related to the use of the MBA method due to the killing of test animals, there are other disadvantages to this technique such as low sensitivity and accuracy. For this reason, in recent years, this method has been replaced mainly by chromatographic techniques.

Currently, the chemical methods are the most efficient and accurate for identifying multiple biotoxins. The HPLC-FLD technique with pre-column hydrogen peroxide and sodium periodate, followed by fluorometric detection, is the reference method for the determination of PSP toxins and is called the Lawrence method [22]. Furthermore, the LC-MS/MS method using HILIC (hydrophilic interaction liquid chromatography) for separating saxitoxins, due to their high polarity, is still being tested for the effective analysis of PSP. Other methods that are less popular for PSP determination are immunological methods like ELISA or receptor binding assay (RBA), which are suitable only for screening purposes [3].

The reference method for domoic acid (DA) toxin group determination in shellfish is HPLC-UV [19]. This assay is based on, first, the extraction of toxins from the sample, usually with an aqueous methanol mixture (50:50, *v*/*v*), and then purification of the extract using a methanol-compatible filter. The purified extract is analyzed with a UV detector. For this purpose, the LC-MS/MS method may also be used, which offers more selectivity and sensitivity through analyzing the mass of the domoic acid molecules and its fragments. This is very important, especially when identifying various isomers of a toxin and confirming its presence in complex seafood matrices [23].

Okadaic acid (OA) and its analogs are analyzed in mollusks through liquid chromatography with tandem mass spectrometry (LC-MS/MS) [22]. The EU reference method is based on the extraction of the okadaic acid group and its esters (OA, DTX), pectenotoxins group (PTX), azaspiracid shellfish poisoning (AZA), and yessotoxins group (YTX) with 100% methanol from homogenized tissue. Then, the extract is filtered and directly analyzed using the LC–MS/MS method in order to determine the presence of toxins. To study the total content of the OA toxins group, an alkaline hydrolysis is required from a methanolic extract prior to LC-MS/MS analysis, with the aim of converting any acylated esters of OA and/or DTXs to the parent OA and/or DTX1 or DTX2 toxins. After that, extracts are filtered and analyzed using LC–MS/MS. The ELISA method is also allowed as an alternative assay for lipophilic toxin determination [24].

In addition to the regulated marine biotoxins, there are also many emerging marine toxins as the effects of globalization and climate change spread rapidly to new areas, which makes them very difficult for identification [1,8,12]. The methods most commonly used for determination of emerging marine biotoxins are based on chromatographic techniques such as LC-MS/MS (brevetoxins, ciguatoxins, and palytoxins), UPLC-MS/MS (cyclic imines), and HILIC-MS/MS (TTX) [1]. The other assays include immunoassays, receptor-binding tests, biosensors, and cell-based approaches, which have been implemented for the screening and quantification analysis of emerging marine toxins. The problem regarding the availability of standards and reference materials is the reason for the limited development of methods for determination of emerging marine biotoxins and, as a consequence, the lack of official methods for these toxins in seafood.

## 3. Occurrence of Marine Biotoxins in Crustaceans

### 3.1. Marine Biotoxins in Crabs

Crabs are omnivorous organisms that might ingest marine toxins from algae or consume other marine organisms that contain toxins. Therefore, they are significant vectors for the higher-level organisms in the food chain, including fish, sea birds, and even humans. In the soft-shell crab industry in the USA, the crabs are usually gutted and cleaned before being sold due to the undesirable bitter taste of the digestive organs [25].

In 2001, at Aveiro lagoon, north-west Portugal, an outbreak of human diarrheic poisonings (DSPs) was revealed after the consumption of numerous cooked green crabs *Carcinus maenas* [13]. Symptoms of poisoning (vomiting, diarrhea, and abdominal pains) appeared 2–3 h after ingestion and persisted over a three-day period [2,3]. In this study, collected crabs were killed by being frozen before the analysis. Then, the samples were thawed, and the crab shell was removed. The edible parts (body meat and most of the viscera) without the leg meat were mixed together. The level of okadaic acid (OA) (mainly in the form of esters) in the edible parts, analyzed using the LC-MS method, was 320 µg/kg of crab viscera. This case of poisoning confirms that crabs *Carcinus maenas*, which eat various species of bivalve mollusks, may be indirect vectors of marine toxins.

In 2002, about 200 persons were poisoned after eating brown crabs *Cancer pagurus* harvested off the south coast of Norway [14,15]. The samples of crabs revealed the presence of 290 μg/kg of OA in the edible tissue [14] and 1500 μg/kg in the brown meat (hepatopancreas) [15]. The source of contamination was blue mussels (*Mytilus edulis*) from the same region, with OA levels reaching up to 4000 µg/kg of mussel meat [14].

In 2008, a study was conducted in Denmark in which green crabs *Carcinus maenas* were fed with raw marine clams containing 2500 mg of okadaic acid equivalents/kg [25]. Each crab was fed one mussel (with an average 2.8 g content of meat) per day for 17 days. The amount of total OA equivalents in the digestive organs was on average 27 times higher than in the crab’s body meat. The highest detected level was 503 and 12 mg/kg of total OA equivalents in digestive organs and body meat, respectively.

The second marine toxin that appears regularly around the Portuguese northern coast is domoic acid (DA) [26]. Mild symptoms of poisoning include vomiting, nausea, and diarrhea, but in more severe cases, neurological symptoms may occur such as dizziness, respiratory difficulty, and coma [1,2,3]. DA was detected in crabs *Carcinus means* but below the USA permitted limit of 30 µg/g of crab viscera [13]. In cooked crabs, a significant reduction in DA was found, which was consistent with the results of the previous studies [27].

Another organism, which may play an important role in ASP in the marine food chain, is the swimming crab *Polybius henslowii* [28]. The crabs were harvested along the Portuguese coast in the summer of 2002. The determined content of DA was high and the maximum content reached 323.1 mg DA/g of crab tissue (in the whole body). The highest level of 576.1 mg DA/g of toxin was in the visceral tissues, while in the remaining tissues, it was 4–12 times lower, which confirms the different distribution of toxins depending on the crab’s organ. In contrast, in *Polybius henslowii* crabs sold during summer at a market in Portugal, DA concentrations detected were close to the legal limit for mussels, i.e., 20 mg/kg [28]. Also, the cooking process was assessed and a reduction in toxins of over 50% during cooking was found.

In Europe, besides Portuguese green crabs (*Carcinus means*) [13] and *Polybius henslowii* [28], Scottish crabs have been reported as vectors for ASP toxins [29].

In Monterey Bay, USA, experimental studies have been conducted based on feeding crabs with razor clams, which contained domoic acid [30]. Experimental studies confirmed that feeding crabs with contaminated mussels leads to toxin accumulation in several species, including Dungeness crabs (*Cancer magister*), rock crab (*Cancer pagurus*), blue crab (*Cancer sapidus*), and stone crab (*Menippe adina*). The aim of this experiment was to understand how the DA toxin accumulates in and is removed from crabs. These studies showed that the toxin accumulates in the crabs’ hepatopancreas, and they do not have an efficient excreting system, which causes a potential risk for marine animals and humans.

Crabs can also cause paralytic shellfish poisoning (PSP) and tetrodotoxin poisoning (TTX) in humans, mainly in North America and Japan [31], but the origin of the toxins has not been established in all cases. Symptoms of PSP and TTX poisoning are similar and cover muscle paralysis, ataxia, dizziness, choking feeling, and shortness of breath [3,32,33]. STXs have been reported most commonly in *Xanthidae* family crabs [34,35]. Xanthid crabs can accumulate toxins in their tissues at high concentrations, which may be dangerous for other consumers [36]. The maximum level of PSP toxin was 6227 μg STXeq/100 g in the Australian Xanthid crabs *Atergatis floridus*, but several samples contained less than 80 μg STXeq/100 g [37]. In contrast, in Japan, *Zosimus aeneus* crabs contained a high level of saxitoxin, amounting to 300 μg STX eq/100 g of samples collected in 1979 [38]. Tetrodotoxin has been found, e.g., in Xanthid crabs from Taiwan (*Demania cultripes*, *Demania toxica*, *Demania reynaudi*, *Lophozozymus incisus*, *Lophozozymus Pictor*, and *Atergatopsis germaini*) [39] and in Horseshoe crab *Carcinoscorpius rotundicauda* from Vietnam [40].

### 3.2. Marine Biotoxins in Lobsters

Lobsters can be a vector for marine biotoxins, mainly for PSP, and may be a serious risk for human health. The main source of toxins for lobsters are bivalve mollusks, especially scallops, which are highly toxic throughout the whole year in many places. Lobsters accumulate harmful toxins in their liver and pancreas after consuming contaminated scallops. High rates of absorption are observed, similar to filter-feeding mussels, which means that lobsters accumulate toxins quickly. There are only a few reports about the occurrence of toxins in European lobsters; most of them come from America and Australia [41,42,43,44,45].

The first study on mussels as an indicator species for lobster was performed in 2012 by Madigan et al. after harmful algal blooms [45]. Mussels are ideal markers due to their high filtration efficiency and rapid biotoxin accumulation. Moreover, there are many well-developed analytical methods for this matrix, and they are easy to collect from harvest areas. The monitoring studies often resulted in the closure of *J. edwardsii* harvests due to the accumulation of a large amount of PSP toxins in their body after consuming contaminated bivalve mollusks. Experimental data have revealed that *J. edwardsii* can collect a large amount of saxitoxin in the liver and pancreas, and this level exceeds the PSP limit in mussels after feeding lobsters with a large amount of highly toxic mussels for four days [46].

In Tasmania, Australia, experimental studies were carried out in which live lobsters were fed with mussels containing PSP toxins collected during the *Alexandrium tamarense* bloom [41]. It was reported that Southern Rock Lobsters (*Jasus edwardsii*) contained PSP toxins at a maximum level of 4050 µg of STX.2HCl eq/kg in their hepatopancreas and in negligible concentrations in other edible tissues. These studies also showed that cooking (steaming or boiling) does not reduce the toxicity and toxin profile, but it decreases the total mass of the hepatopancreas, which reduces the total amount of toxins that could be consumed. In the USA, preventive measures have been taken due to the high risk of PSP after eating lobsters. The Food and Drugs Administration (FDA) does not recommend eating guts from American lobsters, which are considered a delicacy (consumed alone or as an addition to sauces), when high levels of PSP toxins occur on the Atlantic coast [4]. Another monitoring program implemented in New Zealand for *J. edwardsii* confirms the serious risk of lobster poisoning [42]. This program is based on monitoring phytoplankton and toxins in mussels in harvest areas.

**Figure 2:**
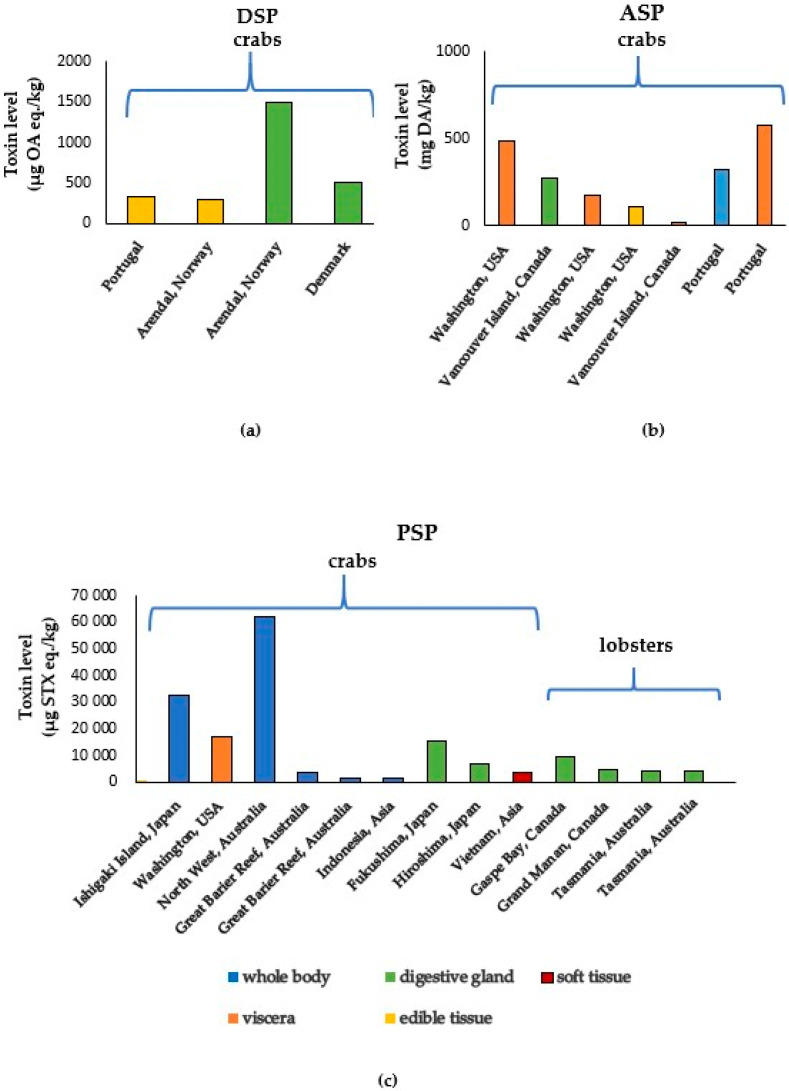
(**a**) DSP, (**b**) ASP, and (**c**) PSP toxin levels reported in crustaceans (crabs and lobsters) from 1980 to 2020 [13,14,15,25,28,31,32,40,41,47,48,49,50,51,52,53,54].

Toxin levels above the regulatory limit were also detected in the hepatopancreas of lobster *Homarus americanus* from eastern Grand Manan Island in Canada, with lower levels (<160 μg STX eq./kg) in the gills and tail [47]. The highest toxin contents identified in this study were 4470, 2435 and 1775 µg STX eq./kg tissue of hepatopancreas, depending on the harvesting site of the harmful algal bloom. This study confirms that scavenging crustaceans, such as *Homarus americanus*, may provide saxitoxins for humans through the consumption of contaminated shellfish, e.g., scallops or blue mussels. The occurrence cases of marine biotoxins in crustaceans like crabs and lobsters are presented in Figure 2.

### 3.3. Marine Biotoxins in Shrimps

The assessment of contaminated shrimp as toxin vectors in the food chain is very important in regard to these organisms consuming phytoplankton throughout their whole life as a valuable source of nutrients, mainly fatty acids [55]. Outbreaks of harmful cyanobacteria and dinoflagellates in farm ponds have caused significant losses of shrimps in farms in Mexico [56,57]. Harmful effects of toxins in shrimps include growth inhibition, impaired behavior, reduction in immunocompetence, and death, which lead to a serious reduction in production [58,59].

To study the toxic influence of the harmful dinoflagellate of *Alexandrium* species on marine organisms, four strains of *Alexandrium* spp. were isolated on the Chinese coast, and their effect on the survival and feeding rates of the brine shrimp *Artemia salina* was analyzed [60]. *A. salina* is a zooplankton organism belonging to crustaceans and occurring in different water reservoirs such as lakes and oceans, and it is a common food for fish and other aquatic organisms [61]. The influence on *A. salina* in each stage of its life cycle—nauplii, metanauplii, and adult—was investigated. The experimental data have shown that *Alexandrium* spp. at a quantity of 2000 cells/mL is lethal to the investigated shrimps *A. salina* and can inhibit their feeding. Paralytic shellfish poisons (PSPs) produced by *Alexandrium* spp. contributed to the death of brine shrimp and restrained their feeding rate. Among the various life stages of *A. salina*, metanauplii was considered most sensitive to the toxic algae.

The first report regarding the assessment of the influence of paralytic shellfish toxins (PSP) from species of *Gymnodinium catenatum* in white leg shrimp (*Litopenaeus vannamei*) was presented in 2008 by Perez-Linares et al. [55]. The shrimps revealed abnormal behavior after the injection of PSP extract. Death occurred in doses above 5.5 μg STX equivalent/g, but lower concentrations resulted in imbalance, paralysis of pereiopods (walking legs) and antennae, abnormal swimming, and abdominal muscle cramps. Main organs such as the heart and brain were seriously damaged, as shown in histological analysis.

McMahon et al. [62] analyzed the influence of tetrodotoxin (TTX) on the larval growing of penaeid shrimp (*Metapenaeus ensis*). In this study, shrimps were injected with a range of concentrations of tetrodotoxin solution, which caused inhibition of the heart in later larval stages. However, no inhibitory effect on heartbeat frequency in earlier juvenile stages of the shrimps was observed.

The first report regarding lipophilic marine biotoxins in brown shrimp (*Crangon crangon*) was described in 2017 by Orellana et al. [63]. The samples were collected close to the coast of the North Sea in Belgium. The mean concentration of OA/DTX-2 toxin in shrimps was low and estimated to be 1.5 µg/kg wet weight, and no other marine biotoxins were found in these crustaceans. This study also showed that shrimps consume small zooplankton organisms such as calanoid copepods and macrobenthic.

## 4. Fish as Non-Bivalve Vectors for Marine Biotoxins

Fish may accumulate marine biotoxins in different ways: directly by eating phytoplankton, by absorbing dissolved toxins through the epithelium after decomposition of algal blooms, or by consuming toxins from the food chain. Fish are particularly sensitive to marine toxins in the early stages of development since fish embryos lack a sufficient enzyme system to remove toxins and have a higher rate of metabolic growth. Fish exposed to harmful phytoplankton are killed by one or a combination of the following processes: by algal neurotoxins, due to irritation/mechanical destruction of the gills through hemolytic substances produced by algal blooms or suffocation caused by the accumulation of mucus in the gills or the lack oxygen in seawater [64].

Ciguatera fish poisoning (CFP) is currently the most common seafood poisoning worldwide, associated with human consumption of subtropical fish that are contaminated with ciguatoxins. There are 50,000–200,000 cases each year of food poisoning after the consumption of fish contaminated with ciguatoxin [65]. The toxin has most frequently been found in the tropical regions of the Pacific, Australia (Brisbane and Great Barrier Reef regions), Central America, and the Caribbean, especially between latitudes 35° north and 35° south. A characteristic feature of ciguatoxin is its progressive accumulation in subsequent steps of the food chain. This leads to the accumulation of this toxin in the consumed fish in amounts that are toxic to humans. Ciguatoxin poisoning manifests with gastrointestinal disorders (vomiting, diarrhea, and abdominal pain), neurological symptoms (headaches and nausea, paresthesia, sensibility disorders, and ataxia), and less common cardiovascular symptoms (bradycardia or tachycardia, hypotension or hypertension) [66]. A concentration of 0.1–5.0 µg/kg of ciguatoxin causes the most cases of CTX poisoning after consumption of contaminated fish [12].

Palytoxin (PTX) is another marine toxin that may occur in fish belonging to mackerel, triggerfish, and filefish species [67]. It is also very dangerous for humans and may cause the contraction of blood vessels and a rapid increase in blood pressure in the heart and lung vessels. Palytoxins are responsible for damaging red blood cells and are characterized by extremely high toxicity to mammals, and the most toxic part of the fish body is the viscera. The syndrome caused by PTX is called clupeotoxism, an uncommon disease connected to the consumption of clupeoid fish, such as herrings, sardines, and anchovies, which has high mortality rates [68]. There have been reports of human near deaths caused by palytoxin poisoning after eating smoked fish (*Decapterus macrosoma*) in Hawaii [69] and after consumption of groupers (*Epinephelus* spp.) or Blue humphead parrotfish (*Scarus ovifrons*) in Japan [70,71].

The first report on the occurrence of okadaic acid in marine fish was in 1996 in the northern Baltic Sea [72]. This toxin was identified in the liver of flounders (*Platichthys flesus*) in the Gulf of Finland. The accumulation of OA was involved in a *Dinophysis acuminata* bloom (520–1960 cells/L). Flounders contained up to 222 µg OA/kg in the liver. Another case is filter-feeder fish *Cetengraulis edentulus* from the Atlantic Ocean that, during a *D. acuminata* bloom, contained 44.7 µg OA/kg in their liver and digestive tract tissues [73]. Off the southern coast of Brazil, Blenny fish *Hypleurochilus fissicornis* contained 59.3 µg OA/kg in free form [74]. There are few reports regarding the accumulation of OA in fish that show that these organisms contain mainly free okadaic acid in viscera for a short time. The following symptoms of OA poisoning have been observed in fish: histological changes in several organs (mainly gills and liver), oxidative stress, abnormal behavior, and in extreme cases, the death of fish [75,76]. Sardines and anchovies belonging to small marine pelagic fish are planktivorous filter feeders and are classified as vectors of domoic acid in the trophic chain during harmful algal blooms [77]. DA levels detected in fish during a study in California were in the range of 131–1815 µg DA/g in anchovy and 169–588 µg DA/g in sardine viscera samples. These fish are food for higher predators such as sea birds and marine mammals like sea lions, and they pose a threat of ASP for these animals. Moreover, fish may also absorb large amounts of marine toxins during intense feeding on bivalves harboring large amounts of toxins.

One of the most dangerous toxins that may occur in fish is tetrodotoxin (TTX). It may cause death in both humans and other mammals, but also birds, many fish species, and other marine animals. The poisoning includes weaker manifestations of gastrointestinal symptoms such as nausea and vomiting or severe poisoning such as bradycardia, respiratory insufficiency, and coma [78]. The level of TTX depends on the body part of fish, with the highest values in the ovary and liver and then in the intestines and skin [79]. According to Commission Regulation (EC) No. 853/2004 establishing specific hygienic rules in the foodstuffs in the European Union, fishery products originating from poisonous fish of the *Tetraodontidae*, *Molidae*, *Diodontidae*, and *Canthigasteridae* families cannot be introduced into the market [6]. Only Japanese law set a regulatory limit of 2 mg equivalent of TTX/kg for tetrodotoxin, while in Europe, these control levels have not been established [78].

Saxitoxin (STX) has been determined along with TTX in several marine and freshwater puffer fish species in countries of the Far East, mainly in Japan [80] and Philippines [81]. STX was also detected in puffer fish from three species: *Sphoeroides nephelus*, with a maximum content of 22, 104 µg STX equiv./100 g tissue, *S. testudineus*, with a max. of 1505 µg STX equiv./100 g tissue, and *S. spengleri*, with a max. content of 1832 µg STX equiv./100 g in Florida, USA [82]. Between 2002 and 2004, hobby harvesting of puffer fish in the Indian River Lagoon resulted in 28 cases of saxitoxin poisoning. The puffer fish contained an average of 1787 µg STX equiv./100 g tissue in the skin. The highest concentrations were also detected in the muscle (1102 µg STX equiv./100 g), gonads (654 µg STX equiv./100 g), gut contents (539 µg STX equiv./100 g), and liver (214 µg STX equiv./100 g). In Japan, studies performed on Starry toado puffer fish (*Arothron firmamentum*) showed that only the ovary and skin of the females were toxic with a range of 5–740 MU/g for STX and <5–30 MU/g for tetrodotoxin (TTX) [83]. Other studies revealed that STXs accumulated in tilapia (*Oreochromis niloticus*) during a toxic cyanobacteria bloom in Brazil but not in high concentrations (around 20–30 µg STX equiv./100 g) [84]. The levels of STX toxins found in these studies were higher than the Acute Reference Dose (ARfD) established at 0.5 μg STX equiv./kg b.w. in shellfish [85]; therefore, the consumption of fish with STX may be very dangerous for human health. The documented cases of marine biotoxins detected in fish are listed in Table 2.

## 5. Conclusions and Future Perspectives

Monitoring the content of marine biotoxins in mollusks and other marine organisms is very important for public health and the fishery industry. The increasing number of novel non-traditional vectors of marine biotoxins should be identified to avoid food poisoning among consumers. Furthermore, the data regarding new organisms which may be vectors for marine biotoxins are crucial for the current risk assessment involved in food poisoning. Despite the fact that the number of poisoning cases with marine biotoxins from various marine organisms worldwide is considerable, there is a lack of precise epidemiological data regarding which of them may cause a risk to public health. The occasional consumption of crabs or lobsters by some consumers raises the problem of possible long-term health effects resulting from the ingestion of low doses of biotoxins in these foods. In contrast, the detection of large amounts of marine biotoxins in fish and seafood may cause economic losses for the fishery and aquaculture industries due to potential fishery closures. Marine biotoxin monitoring programs in European Union legislation are facing new problems due to the growing demand for fish and seafood worldwide. Also, new species of seafood, so far eaten in small amounts, are becoming increasingly popular among consumers.

Taking into account the different anatomical structure, habitat, and feeding habits of new toxin vectors like crabs, shrimps, lobsters, and fish, detailed regulations should be introduced to ensure the safety of consuming these organisms. They need to include issues such as the classification of production areas, sampling frequency and size, and problems with the determination of toxins in different matrices. In reference to the last issue, further studies are needed to better describe the mechanisms of action of toxins in new matrices like crustaceans and fish to develop methods for the purification of samples and to study the matrix effects. In summary, future studies on monitoring marine biotoxins in crustaceans and fish should focus on development and improvement of analytical techniques to determine new toxins and their derivatives in new matrices, evaluating a wider range of organisms as potential vectors, and assessing the long-term influence of biotoxins on marine life and humans.

## Figures and Tables

**Table 1:** European Union (EU)-regulated marine toxins in live bivalve mollusks and emerging marine toxins.

Marine Biotoxin	Group	Regulatory Limit	Detection Method
**Regulated marine toxins in the EU**			
Saxitoxins	STx	800 µg STX eq.2-HCl/kg	HPLC-FLD *
Domoic acid	DA	20 mg DA/kg	HPLC-UV *
Okadaic acid	OA	160 µg OA eq./kg	LC-MS/MS *
Azaspiracids	AZAs	160 µg AZA eq./kg	LC-MS/MS *
Yessotoxins	YTXs	3.75 mg YTX eq./kg	LC-MS/MS *
**Emerging marine toxins (not regulated in the EU)**			
Brevetoxins	BTx	NE **	LC-MS/MS
Ciguatoxins	CTx	NE **	LC-MS/MS
Cyclic imines	CIs	NE **	UPLC-MS/MS, LC-MS/MS,
Tetrodotoxins	TTx	NE **	HILIC-MS/MS, UPLC-MS/MS
Palytoxins	PTXs	NE **	LC-MS/MS

* Official method; ** NE, not established.

**Table 2:** Highest toxin levels reported in fish during 1995 to 2020.

Species	Toxin	Toxin Concentration	Analyzed Tissue	Area	Year	Reference
Southern Puffer Fish (*Sphoeroides nephelus*)	PSP	221,040 µg STXeq./kg	Gonads	Florida, Indian River Lagoon	2004	[82]
Southern Puffer Fish (*Sphoeroides nephelus*)	PSP	201,060 µg STXeq./kg	Muscle tissue	Florida, Indian River Lagoon	2002–2006	[82]
Southern Puffer Fish (*Sphoeroides nephelus*)	PSP	172,360 µg STXeq./kg	Skin	Florida, Indian River Lagoon	2002–2006	[82]
Southern Puffer Fish (*Sphoeroides nephelus*)	PSP	136,660 µg STXeq./kg	Gut contents	Florida, Indian River Lagoon	2002–2006	[82]
Southern Puffer Fish (*Sphoeroides nephelus*)	PSP	47,020 µg STXeq./kg	Skin	Florida, Tequesta	2002–2006	[82]
Southern Puffer Fish (*Sphoeroides nephelus*)	PSP	33,260 µg STXeq./kg	Skin	Florida, Tampa Bay	2002–2006	[82]
Southern Puffer Fish (*Sphoeroides nephelus*)	PSP	12,830 µg STXeq./kg	Gonads	Florida, Jacksonville	2002–2005	[82]
Checkered Puffer Fish (*Sphoeroides testudineus*)	PSP	15,050 µg STXeq./kg	Skin	Florida, Tequesta	2002–2006	[82]
Checkered Puffer Fish (*Sphoeroides testudineus*)	PSP	3080 µg STXeq./kg	Skin	Florida, Indian River Lagoon	2002–2006	[82]
Checkered Puffer Fish (*Sphoeroides testudineus*)	PSP	2650 µg STXeq./kg	Gut contents	Florida, Tequesta	2002–2006	[82]
Checkered Puffer Fish (*Sphoeroides testudineus*)	PSP	1890 µg STXeq./kg	Gonads	Florida, Indian River Lagoon	2002–2006	[82]
Bandtail puffer fish (*Sphoeroides spengleri*)	PSP	18,320 µg STXeq./kg	Skin	Florida, Indian River Lagoon	2002–2006	[82]
Bandtail puffer fish (*Sphoeroides spengleri*)	PSP	17,780 µg STXeq./kg	Muscle	Florida, Tequesta	2002–2006	[82]
Bandtail puffer fish (*Sphoeroides spengleri*)	PSP	15,340 µg STXeq./kg	Skin	Florida Keys	2002–2006	[82]
Bandtail puffer fish (*Sphoeroides spengleri*)	PSP	14,280 µg STXeq./kg	Gut contents	Florida, Indian River Lagoon	2002–2006	[82]
Starry toado puffer fish (*Arothron firmamentum*)	PSP	133,200 µg STXeq./kg *	Ovary	Japan, Iwate	2000	[83]
Starry toado puffer fish (*Arothron firmamentum*)	PSP	27,360 µg STXeq./kg *	Ovary	Japan, Oita Prefecture	1995	[83]
Tilapia (*Oreochromis niloticus*)	PSP	300 µg STXeq./kg	Muscle	Brazil, Sao Paulo	2005	[84]
Starry toado puffer fish (*Arothron firmamentum*)	TTX	6600 µg TTXeq./kg **	Skin	Japan, Oita Prefecture	1995	[85]
Torafugu puffer fish (*Takifugu Rubripes*)	TTX	880,000 µg TTXeq./kg	Liver	Japan, Saitama Prefecture	2000	[86]
Torafugu puffer fish (*Takifugu Rubripes*)	TTX	352,000 µg TTXeq./kg	Ovary	Japan, Saitama Prefecture	2000	[86]
Silver cheeked puffer fish (*Lagocephalus sceleratus*)	TTX	13,480 µg TTXeq./kg	Liver	Northern Cyprus Sea	2017–2018	[87]
Amberjack (*Seriola* spp.)	CTX	6.439 µg CTX1Beq./kg	Liver	Canary Islands	2016–2019	[88]
Black Moray Eel (*Muraena helena*)	CTX	6.062 µg CTX1Beq./kg	Liver	Canary Islands	2016–2019	[88]
Flounder (*Platichthys flesus*)	DSP	222 µg OA/kg	Liver	Baltic Sea, Gulf of Finland	1996	[72]
Atlantic Anchoveta (*Cetengraulis edentulus*)	DSP	44.7 µg OA/kg	Guts + liver	Brazil, Paranagua	2011	[73]
Blenny fish (*Hypleurochilus fissicornis*)	DSP	59.3 µg OA/kg	Viscera	Brazil, Santa Catarina	2016	[74]
Anchovy (*Engraulis mordax*)	ASP	1815; 1175; 1600; 1050; 507; 288 mg DA/kg	Viscera	California, Monterey Bay	2000	[77]
Sardines (*Sardinops sagax*)	ASP	588; 558; 551; 386; 279; 216; 169 mg DA/kg	Viscera	California, Monterey Bay	2000	[77]

* Mouse Units were converted into STX equivalents, considering 1MU = 0.18 µg STX. ** Mouse Units were converted into TTX equivalents, considering 1MU = 0.22 µg TTX.

## Data Availability

No new data were created or analyzed in this study.

## Abbreviations

The following abbreviations are used in this manuscript:

ASP	Amnesic shellfish poisoning
AZP	Azaspiracid shellfish poisoning
BTX	Brevetoxin
CTX	Ciguatoxin
DSP	Diarrhetic shellfish poisoning
DTX	Dinophysistoxin
HILIC-MS/MS	Hydrophilic interaction liquid chromatography with tandem mass spectrometry
HPLC-FLD	High-performance liquid chromatography with fluorescence detection
HPLC-UV	High-performance liquid chromatography with ultraviolet detection
LC-MS/MS	Liquid chromatography with tandem mass spectrometry
MBA	Mouse bioassay
PSP	Paralytic shellfish poisoning
PTX	Palytoxin
TTX	Tetrodotoxin
UPLC-MS/MS	Ultra-performance liquid chromatography with tandem mass spectrometry
YTX	Yessotoxoin
